# PAX4 Defines an Expandable β-Cell Subpopulation in the Adult Pancreatic Islet

**DOI:** 10.1038/srep15672

**Published:** 2015-10-27

**Authors:** Petra I. Lorenzo, Esther Fuente-Martín, Thierry Brun, Nadia Cobo-Vuilleumier, Carmen María Jimenez-Moreno, Irene G. Herrera Gomez, Livia López Noriega, José Manuel Mellado-Gil, Alejandro Martin-Montalvo, Bernat Soria, Benoit R. Gauthier

**Affiliations:** 1 Pancreatic Islet Development and Regeneration Unit, Department of Stem Cells, CABIMER-Andalusian Center for Molecular Biology and Regenerative Medicine, Seville, Spain; 2 Department of Cell Physiology and Metabolism, University of Geneva, Geneva, Switzerland; 3 Cellular Therapy of Diabetes Mellitus and its Complications, Department of Stem Cells, CABIMER-Andalusian Center for Molecular Biology and Regenerative Medicine, Seville, Spain;; 4 CIBERDEM, Instituto Carlos III, Madrid, Spain

## Abstract

PAX4 is a key regulator of pancreatic islet development whilst in adult acute overexpression protects β-cells against stress-induced apoptosis and stimulates proliferation. Nonetheless, sustained PAX4 expression promotes β-cell dedifferentiation and hyperglycemia, mimicking β-cell failure in diabetic patients. Herein, we study mechanisms that allow stringent PAX4 regulation endowing favorable β-cell adaptation in response to changing environment without loss of identity. To this end, PAX4 expression was monitored using a mouse bearing the enhanced green fluorescent protein (GFP) and cre recombinase construct under the control of the islet specific pax4 promoter. GFP was detected in 30% of islet cells predominantly composed of PAX4-enriched β-cells that responded to glucose-induced insulin secretion. Lineage tracing demonstrated that all islet cells were derived from PAX4^+^ progenitor cells but that GFP expression was confined to a subpopulation at birth which declined with age correlating with reduced replication. However, this GFP^+^ subpopulation expanded during pregnancy, a state of active β-cell replication. Accordingly, enhanced proliferation was exclusively detected in GFP^+^ cells consistent with cell cycle genes being stimulated in PAX4-overexpressing islets. Under stress conditions, GFP^+^ cells were more resistant to apoptosis than their GFP^-^ counterparts. Our data suggest PAX4 defines an expandable β-cell sub population within adult islets.

During embryogenesis, both the exocrine and endocrine compartment of the pancreas arises through the interplay of numerous transcription factors that will temporally and spatially bestow the fate of the various cell lineages^1^. Among these, the paired homeodomain nuclear factor Pax4 is indispensable for the generation of islet cell progenitors and subsequent β-cell maturation. Although detectable, PAX4 expression in adult islet β-cells is low as compared to its embryonic expression^2^. In contrast, aberrantly high expression levels for this transcription factor are detected in human insulinomas, lymphomas, head and neck squamous cell carcinomas as well as in breast cancer cells^3^,^4^,^5^. A distinctive attribute of *Pax4* is that mutations and polymorphisms in this gene are associated with both Type 1 and 2 Diabetes Mellitus (T1DM and T2DM), as well as with maturity onset diabetes of the young (MODY) in several ethnic populations^2^, with a strong prominence in the Asian population^6^,^7^,^8^,^9^,^10^,^11^. *Pax4* gene variations also predispose to Ketosis-prone diabetes in populations of West African ancestry^12^. Paradoxically, *Pax4* polymorphisms were also linked to longevity in the Korean population^13^.

Since a hallmark of both T1DM and T2DM, independent of etiology, is the gradual loss of the functional insulin-producing β-cell mass, we and others have demonstrated that PAX4 is not only essential for islet development^14^ but also for survival and expansion of adult β-cells^15^,^16^. In mice, conditional overexpression of PAX4 in β-cells was shown to protect animals against streptozotocin (STZ)-induced hyperglycemia and isolated islets against cytokines induced apoptosis. In contrast, animals expressing the diabetes-linked mutant variant R121W (R129W in mice) were more susceptible to develop hyperglycemia and β-cell death upon STZ treatment. Interestingly, sustained expression of PAX4 *in vivo* resulted in loss of islet structure and insulin secretion with the concomitant appearance of a BrdU^+^/PDX1^+^/INSULIN^−^ cell subpopulation suggesting dedifferentiation of β-cells that potentially acquire a proliferative phenotype^17^. Intriguingly, β-cell dedifferentiation characterized by the loss of INSULIN granules and re-expression of the pancreatic endocrine progenitor marker NGN3 was also recently reported in various animal models of T2DM^18^,^19^. Restoration of functional β-cells was achieved upon normalization of blood glucose levels using insulin therapy indicating that the hyperglycaemic milieu favoured survival through loss of β-cell identity at the expense to attempt rescuing glucose homeostasis^19^. The potential implication of PAX4 in this process was recently connoted through data demonstrating that transcript levels for this factor were increased in islets isolated from T2DM donors^20^. The correlation between PAX4 expression levels and the phenotypic state of β-cells led us to characterize *in vivo* PAX4 regulation within the islets under various physiological and pathophysiological conditions. To this end, we took advantage of a transgenic mouse model expressing both the enhanced green fluorescence protein (GFP) and the *Cre* recombinase under the control of the pancreatic islet specific *Pax4* gene promoter region^21^ to monitor in real time the endogenous expression pattern of PAX4 under various metabolic conditions. We demonstrate that within mature islets endogenous PAX4 marks predominantly a subset of islet β-cells, which on one hand is more susceptible to expansion in response to increased insulin demands such as pregnancy, while on the other hand, is more resistant to stress-induced apoptosis.

## Results

### PAX4 is heterogeneously expressed within adult mice pancreatic islet cells

**Figure 1:**
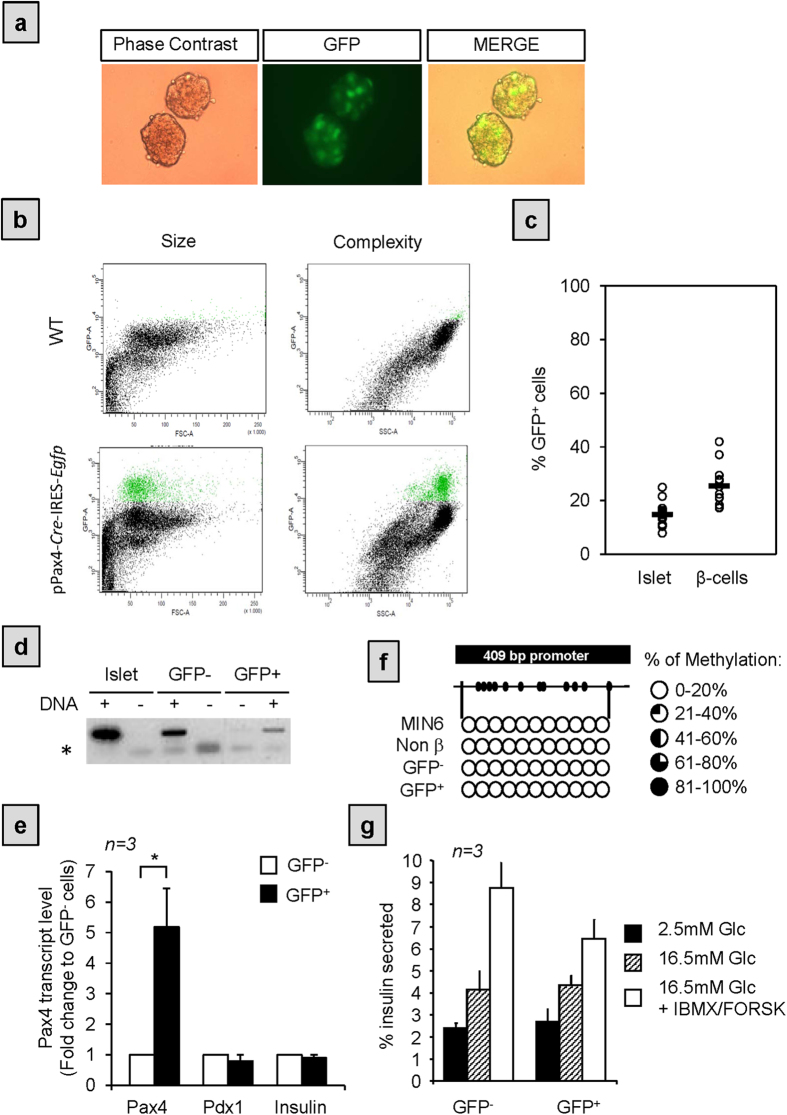
GFP is expressed in a subset of islet cells that are enriched in Pax4 mRNA. (**a)** Phase contrast and fluorescence microscopy images of intact live islets isolated from pPax4-*Cre*-IRES-*Egfp* mice showing GFP fluorescence only in a subset of islet cells. (**b**) Representative cytometer plots corresponding to dispersed adult islets from C57BL/6 wild type (WT)(upper panels) and pPax4-*Cre*-IRES-*Egfp* (lower panels) mice. Plots represent GFP fluorescence (Y axis) vs. cell size (X axis left plots) or cell complexity (X axis right plots). GFP expressing cells (green) display similar size and complexity as the β-cells. (**c**) Quantification of the cytometry analysis. Each dot corresponds to an independent analysis using islet pools from at least 3 animals and presented either per islet or per β-cell population. (**d**) Agarose gel electrophoresis of PCR-amplified *gfp* fragment confirming transgene insertion in both GFP^+^ and GFP^−^ islet cell populations (DNA +). Negative controls for PCR are indicated as DNA -. Non-specific primer-dimers are denotes as *. (**e**) Fold change in Pax4, Pdx1 and Insulin transcript levels in sorted GFP^+^ cells relative to the levels detected in the GFP^−^ fraction. *T-Student: p < 0.05. (**f**) Bisulfite sequencing analysis of the 409 bp *pax4* gene promoter region. The analysis was performed in parallel in MIN6 cells and in the different sorted islet cell populations, showing in all of them less than 20% methylation for each of the CpG sites. (**g**) Insulin secretion was assessed in 30 min static incubations in response to 2.5 mM (black bars) and 16.5 mM glucose alone (hashed bars) or in combination with 1 μM IBMX/forskolin (white bars). Insulin released was quantified by ELISA and expressed as a percentage of total cellular insulin content. Results are the mean ± SE from 3 independent experiments conducted on either GFP^+^ or GFP^−^ pooled fractions, performed in duplicates.

Previous studies performed *in vitro* as well as *in vivo* have shown that acute PAX4 expression is important for β-cell survival and/or expansion while chronic expression triggers dedifferentiation and tumour formation^3^,^4^,^5^,^15^,^17^,^22^. In order to elucidate the mechanism by which β-cells fine tune PAX4 expression without shedding identity and ultimately inducing hyperglycemia we took advantage of a transgenic mouse model expressing both the *Gfp* and the *Cre* cDNAs under the transcriptional control of an optimal *Pax4* gene promoter sequence (pPax4-*Cre*-IRES-*Egfp* mice)^23^. The latter minimal promoter fragment was shown to direct islet specific expression of the *pax4* gene mimicking the full-length promoter^21^,^24^. The pPax4-*Cre*-IRES-*Egfp* transgenic animals display normal islet architecture as well as glucose homeostasis during their life span thereby providing a powerful model to monitor endogenous levels of PAX4 through GFP fluorescence. Live imaging of whole islets derived from these mice revealed that only a fraction of cells emitted detectable levels of GFP fluorescence, revealing potential cell heterogeneity in PAX4 expression (Fig. 1a). To validate this premise, cell suspensions were prepared from islets derived from either wild type C57BL/6 or pPax4-*Cre*-IRES-*Egfp* mice and the various cell populations were discriminated by size or complexity against fluorescence intensity using flow cytometry (Fig. 1b). This approach revealed that 14.79 ± 4.06% of total islet cells expressed GFP whereas 25.48 ± 6.57% of the total β-cell population expressed GFP (Fig. 1c). Despite GFP expression only in a subset of islet cells, genomic amplification confirmed transgene integration in both GFP^+^ and GFP^−^ subpopulations reinforcing the premise that the *Pax4* gene promoter driving GFP expression was only active in a subpopulation of islet cells (Fig. 1d). Consistent with the latter, endogenous *Pax4* transcript levels were 5-fold enriched in the GFP^+^ cell subpopulation (Fig. 1e). Of note, the average Ct value of Pax4 in GFP^+^ cells was approximately 29 while the Ct values for the Cyclophilin control gene was 22. Henceforth, this cell population will be denoted as GFP/PAX4^+^. In order to further characterize this GFP/PAX4^+^ subpopulation, we next assess expression levels of the β-cell markers Pdx1 and Insulin. Transcript levels for either gene remained relatively constant in both subpopulations (Fig. 1e). Consistent with the latter, immunostaining of PDX1 revealed expression of the transcription factor in the vast majority of GFP/PAX4^+^ cells as well as in the GFP^−^ cells (Supplementary Fig. S1). As DNA methylation is associated with transcriptional silencing of *Pax4*^3^,^20^, we assessed the methylation status of the 11 GpC sites found within the 409 bp promoter region (Fig. 1f). This region was hypomethylated in both the GFP/PAX4^+^ and GFP^−^ subpopulations as well as in MIN6 cells that expressed high levels of PAX4 (Fig. 1f). Thus the methylation profile cannot account for differential GFP expression within the two subpopulations. Interestingly, both GFP/PAX4^+^ and GFP^−^ subpopulations displayed similar insulin secretion in response to glucose alone or in combination with IBMX/forskolin indicating that GFP/PAX4-expressing cells retained functionality under normal physiological conditions (Fig. 1g). Taken together these data suggest that PAX4 expression is restricted to a subpopulation of cells phenotypically and functionally similar to β-cells.

### PAX4 expression is gradually confined to a subset of islet cells with age, correlating with decreased proliferation

**Figure 2:**
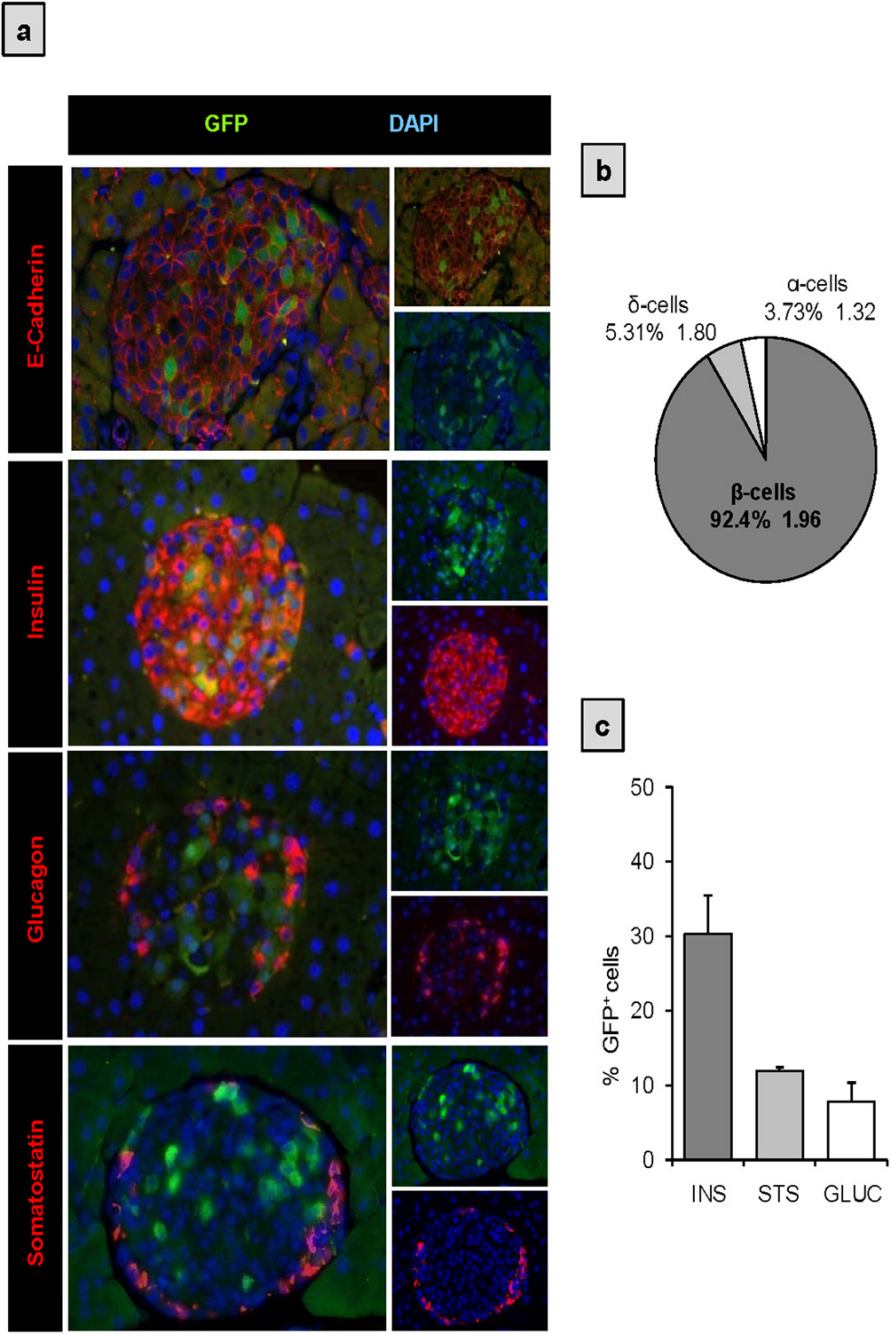
GFP expression predominantly co-localizes with insulin-producing β-cells in adult islets. (**a**) Immunohistochemistry analysis of paraffin sections from adult pPax4-*Cre*-IRES-*Egfp* mice pancreas. Representative microscopy images for co-immunofluorescent labeling of GFP (green) together with E-CADHERIN (for cellular delimitation) or with the pancreatic islet cell markers INSULIN (red), GLUCAGON (red) or SOMATOSTATIN (red). Nuclei counterstaining was performed using DAPI (blue). (**b**) Quantitative analysis (average ± SE) of the GFP^+^ cell population composition. (**c**) Quantification of the percentage of INSULIN^+^, GLUCAGON^+^ and SOMATOSTATIN^+^ cells that co-express GFP (average ± SE). Sections (2 to 4) from 3 to 5 independent animals with an average of 15 islets and 1400 cells per animal were used for quantifications.

To assess in more detail the spatial distribution as well as to determine the specific cell types expressing GFP within islets, we performed immunohistochemistry analysis of pancreas sections derived from pPax4-*Cre*-IRES-*Egfp* adult mice (Fig. 2). Co-immunostaining using an antibody against the cell membrane protein E-CADHERIN along with an anti-GFP sera, clearly established the presence of a GFP/PAX4^+^cell subset randomly distributed within islets (Fig. 2a). Analysis of GFP co-expression with markers of different islet cell types revealed that more than 90% of the GFP/PAX4^+^ subpopulation were insulin-expressing cells (Fig. 2a,b) and comprised approximately 30% of the total β-cell population (Fig. 2c). However, 3.73 ± 1.32% and 5.31 ± 1.80% of GFP/PAX4^+^ cells co-immunostained for GLUCAGON and SOMATOSTATIN respectively (Fig. 2a,b), representing roughly 6% and 10% of the total α- and δ-cell populations (Fig. 2c).

**Figure 3:**
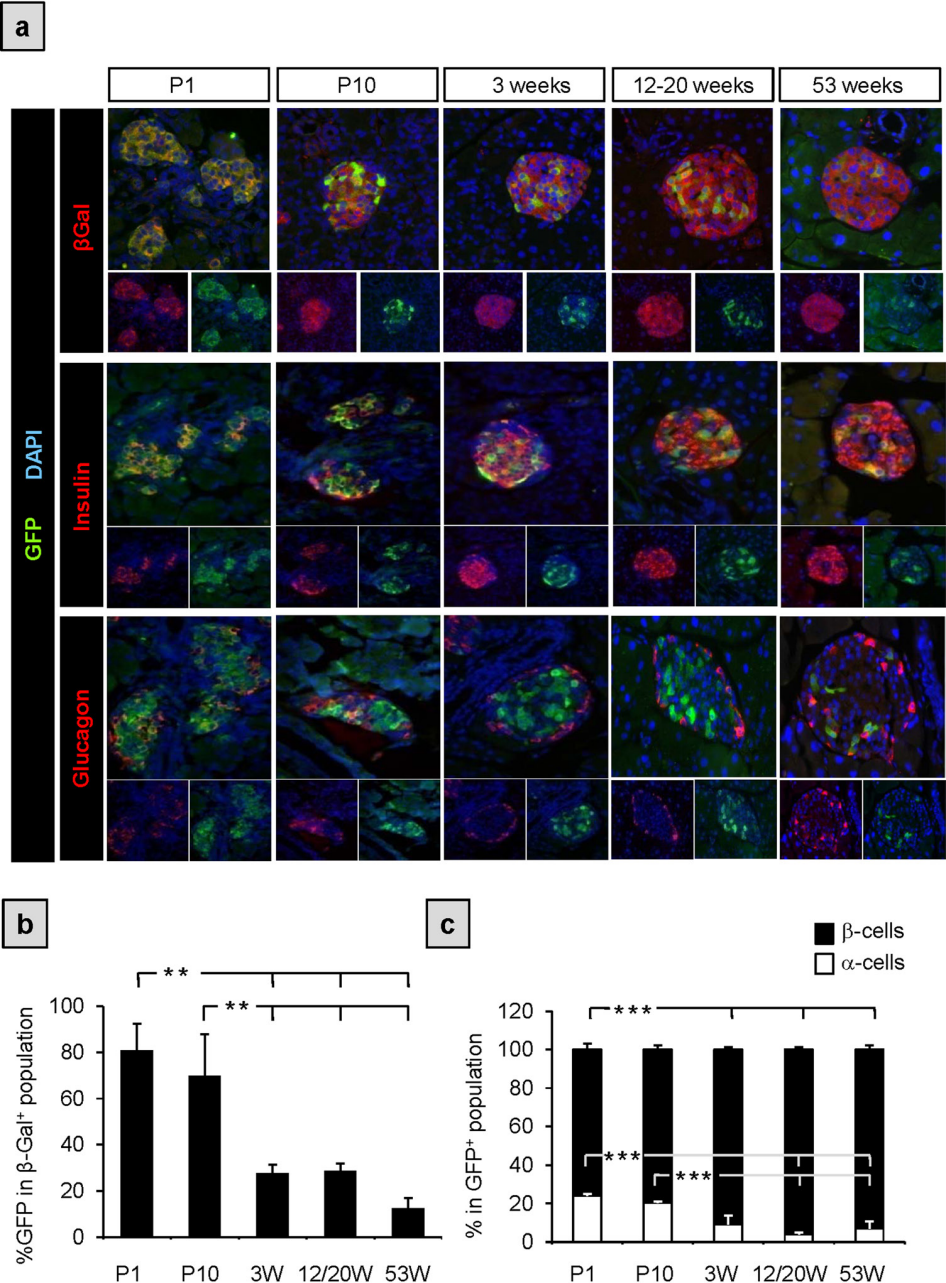
Lineage tracing analysis reveals an age dependent decrease in the GFP/Pax4^+^subpopulation. (**a**) Representative images from IHC of paraffin sections of pPax4-*Cre*-IRES-*Egfp*X*Rosa26*;lox-stop-lox;*LacZ* mice at the indicated ages. Co-immunodetection of GFP (green) along with β-GAL (red, upper panels), INSULIN (red middle panels) or GLUCAGON (red lower panels). Nuclei counterstaining was performed using DAPI (blue). (**b**) Quantification of β-Gal cells that co-express GFP at the indicated ages (average ± SE). (**c**) Quantitative analysis (average ± SE) of GFP^+^ cells within the β- and α-cell populations. Sections (2 to 4) from 3 to 5 animals with an average of 10 islets and 900 cells per animal were used for quantifications. **ANOVA:p < 0.01; ***ANOVA:p < 0.001.

**Figure 4:**
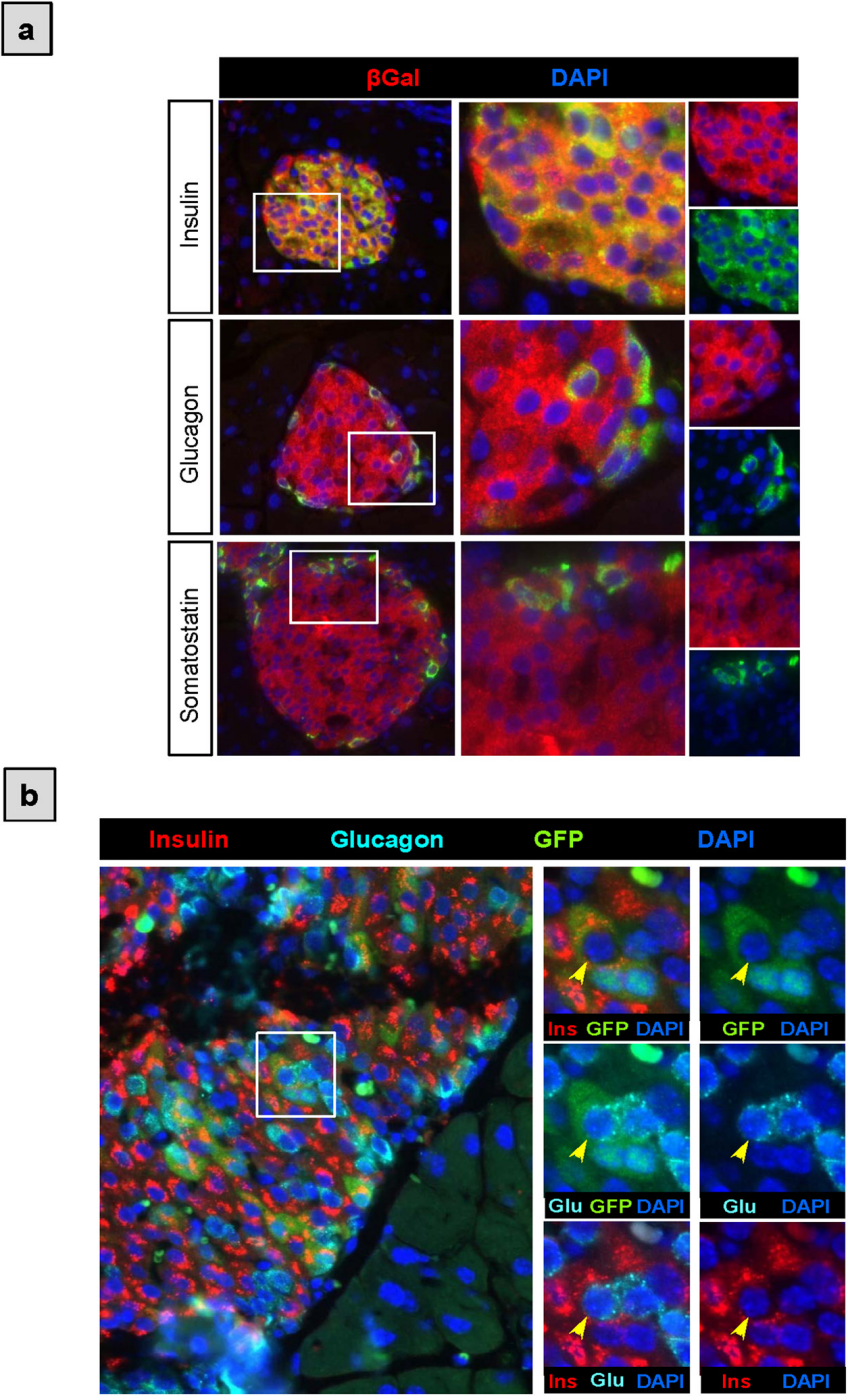
GFP/PAX4^+^ cells contribute to all endocrine cell progeny. (**a**) Co-immunohistochemical analysis of β-Gal (red) along with INSULIN, GLUCAGON and SOMATOSTATIN (green) on pancreatic paraffin sections from adult pPax4-*Cre*-IRES-*Egfp*X*Rosa26*;lox-stop-lox;*LacZ* mice. Nuclei were stained with DAPI (blue). (**b**) Triple-immunostaining for INSULIN (red) GLUCAGON (cian) and GFP (green). Inset with yellow arrows delineate a representative GFP/PAX4^+^ cell co-expressing INSULIN and GLUCAGON. Nuclei were stained with DAPI (blue).

To determine the fate of GFP/PAX4^+^ cells after birth, we performed lineage-tracing experiments. To this end, pPax4-*Cre*-IRES-*Egfp* mice were crossed to mice carrying the *Rosa26*;lox-stop-lox;*LacZ* reporter (R26R). In double transgenic animals (pPax4-*Cre*-IRES-*Egfp*;R26R), Pax4 promoter driven expression of CRE should result in the excision of the lox-stop-lox cassette hence permanently activating the *LacZ* gene in all PAX4-expressing cells along with their downstream progeny. Accordingly, β-Galactosidase (β-Gal) staining was detected in all islet cells at postnatal day 1 (P1) and remained constant until the age of 53 weeks (Fig. 3a, upper panels). Reciprocally, INSULIN, GLUCAGON and SOMATOSTATIN co-localized with β-Gal (Fig. 4a) substantiating previous findings that PAX4 expression is induced early on during development in endocrine progenitors that give rise to all islet cells^25^. These results also demonstrate that despite possible limitations of minimal promoter studies, the *pax4* gene promoter used in this transgenic mouse model is sufficient to recapitulate endogenous PAX4 expression during pancreas development and in adulthood. In contrast to β-Gal staining throughout the whole islet, GFP expression was detected in approximately 70 to 80% of these cells at P1 as well as P10 and was thereafter restricted to 30% of β-Gal^+^ cells by the age of 3-weeks (Fig. 3a,b). This fraction of GFP/PAX4^+^ cells remained relatively constant during adulthood until the age of 1 year (53 weeks) at which point GFP expression was further reduced to 15% of β-Gal^+^ cells (Fig. 3a,b). Noteworthy, this decrease in GFP/PAX4^+^ population correlated with the age-dependent reduction in the ratio of proliferating islet cells, from 6.37 ± 1.97% at P10 to 0.81 ± 0.27% in adult mice and further decrease to 0.6 ± 0.1% in aged animals (data not shown). As expected, INSULIN^+^ cells encompassed the majority of the GFP/PAX4^+^ population during adulthood (Fig. 3a,c). However during early postnatal stages, P1 and P10, the percentage of GFP/PAX4^+^ cells that co-express GLUCAGON was approximately 20%, decreasing with maturity to approximately 3% in adult animals (Fig. 3a,c). Of note, we also detected rare glucagon/insulin co-expressing GFP/PAX4^+^ cells (Fig. 4b) indicative of either an early bi-hormonal precursor pool or trans-differentiation of GLUCAGON/GFP/PAX4^+^ cells towards a β-cell phenotype. Together, these lineage-tracing experiments show that PAX4 gives rise to all endocrine cells during development and that its expression subsequently becomes restricted to a fraction of islet cells predominantly composed of β-cells with residual expression in some α- and δ-cells as well as in rare bi-hormonal (GLUCAGON/INSULIN) expressing cells.

### PAX4 primes cell cycle genes correlating with the preferential expansion of the GFP/PAX4^+^ cell subset during pregnancy

**Figure 5:**
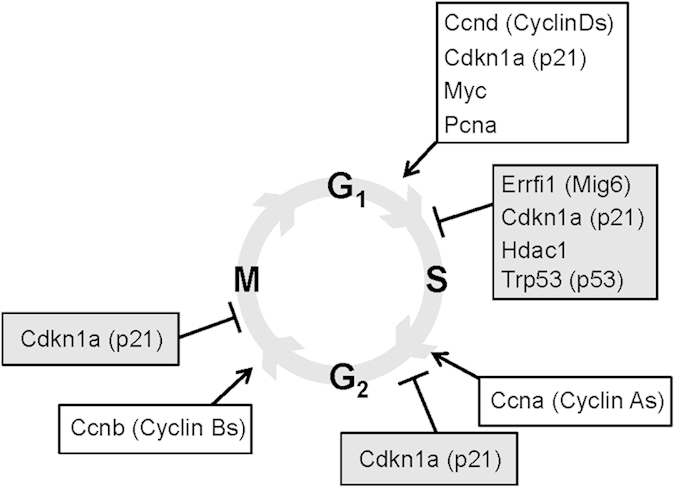
PAX4 regulates cell cycle genes. Schematic representation of PAX4 regulated cell cycle activators (white boxes) and inhibitors (grey boxes) as determined by RNA microarray analysis performed on PAX4 overexpressing islets (GSE62846).

**Figure 6:**
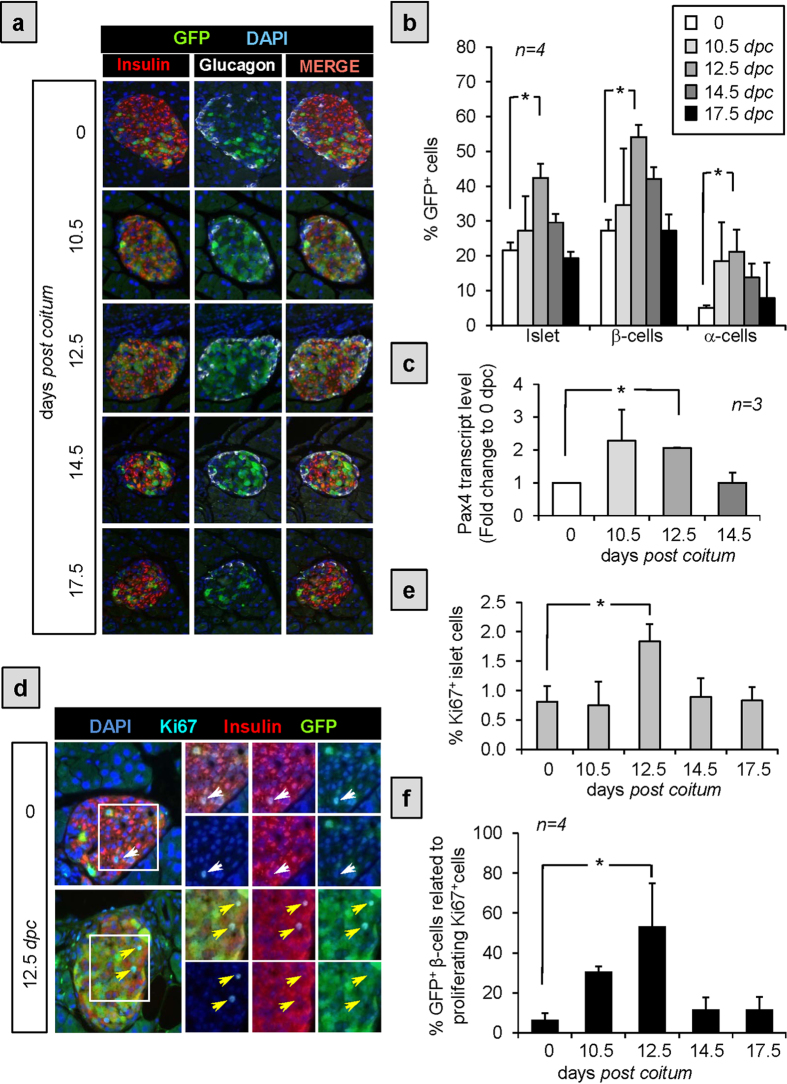
The GFP/PAX4^+^subpopulation is transiently increased during pregnancy. (**a**) Representative composite images of pancreas sections obtained from pregnant pPax4-*Cre*-IRES-*Egfp* females that were co-immunostained for GFP (green), INSULIN (red) and GLUCAGON (white). Nuclei counterstaining was performed using DAPI (blue). (**b**) Cell quantification of percent GFP/PAX4^+^ cells distributed within the total islet cell, β-cell and α-cell population. (**c**) Fold change in endogenous Pax4 mRNA levels measured by qt-RT-PCR in whole islets from pregnant C57BL/6 mice. (**d**) Representative composite images of pancreas sections obtained from control and 12.5 *dpc* pPax4-*Cre*-IRES-*Egfp* females that were triple-immunostained for GFP (green), INSULIN (red) and KI67 (cian). Nuclei counterstaining was performed using DAPI (blue). Insets depict KI67^+^/GFP^−^ cells (white arrow) and KI67^+^/GFP^+^ cells (yellow arrow). (**e**) Quantitative analysis (average ± SE) of the proliferative, Ki67^+^ cells during pregnancy. (**f**) Cell quantification represented as the percentage of GFP^+^β-cells among the total amount of proliferating KI67^+^cells. Data show the mean ± SE of 3 to 5 animals with an average of 15 islets and 1200 cells per animal were used for quantifications.*ANOVA:p < 0.05 as compared to day 0.

The fraction of replicative β-cells drastically decreases with age correlating with the observed decline in GFP/PAX4^+^cells (Fig. 3). The association of these two events combined with our previous finding that PAX4 induces cell replication^15^ prompt us to investigate whether the GFP/PAX4^+^ subpopulation could represent the β-cell replicative unit of the islet. A recent gene expression analysis conducted on PAX4 overexpressing islets revealed a significant functional enrichment in the cell cycle pathway. A more detailed *in silico* gene analysis within this pathway disclosed that PAX4 induced the expression of both cell cycle activators such as CYCLIN A2, B2, D1 and D3, CMYC, HDAC and PCNA as well cell cycle inhibitors such as P21, P53, OTUB2 and ERRFI1 (Table 1). These data suggest that PAX4 defines a proliferation permissive β-cell subpopulation primed to expansion only under conditions that alleviate cell cycle brakes (Fig. 5). To validate this hypothesis, we assessed the replicative capacity as well as expansion of the GFP/PAX4^+^ islet cell subset during pregnancy, a physiological condition associated with intense β-cell replication^26^. Immunohistochemical analysis, using antibodies against GFP, INSULIN and GLUCAGON, were performed on pancreas sections derived from pregnant pPax4-*Cre*-IRES-*Egfp* mice sacrificed at 10.5, 12.5, 14.5 and 17.5 days *post coitum* (*dpc*) (Fig. 6). In agreement with previous studies^27^, islet-cell proliferation peaked at 12.5 *dpc* (Fig. 6e). Consistent with the latter, a significant transient increase in the overall number of GFP/PAX4^+^ cells was observed, reaching 45 ± 3% of total islet cell population by 12.5 *dpc* and decreasing thereafter to 19 ± 1% by 17.5 *dpc* (Fig. 6a,b). Accordingly, 52 ± 3% of β-cells were GFP/PAX4^+^ while 20 ± 6% of α-cells expressed GFP by 12.5 *dpc* (Fig. 6a,b). Consistent with the expansion of the GFP/PAX4^+^ cell subpopulation, a transient increase in endogenous Pax4 transcript levels was also detected in whole islets of pregnant mice reaching maximal levels of 2-fold at 12.5 *dpc* as compared to islets isolated from non-pregnant females (Fig. 6c). We next assessed cell proliferation within the GFP/PAX4^+^ and GFP^−^ subpopulations. To this end, immunostaining for the proliferation marker Ki67 along with GFP and INSULIN was performed on sections of pregnant mice pancreas (Fig. 6d). In adult non-pregnant mice, 6 ± 3% of Ki67^+^ cells were insulin expressing GFP/PAX4^+^ (Fig. 6f) while 58 ± 6% were GFP^−^ β-cells. However, in pregnant mice by 12.5 *dpc* the number of GFP/PAX4^+^ β-cells dramatically increased to 55 ± 20% of the Ki67^+^ proliferating cell population (Fig. 6f) whereas replication of GFP^−^ β-cells remained relatively constant (data not shown). This increase was transient as by 17.5 *dpc* proliferation of the GFP/PAX4^+^ population regressed to control values. Taken together our results suggest that although the fraction of proliferating GFP/PAX4^+^ β-cells is lower than that of the GFP^−^ β-cell fraction in control animals, GFP/PAX4^+^ β-cells are more permissive to proliferation and expansion upon conditions stimulating β-cell replication such as during pregnancy.

### GFP/PAX4^+^ islet cells are more resistant to stress-induced apoptosis

**Figure 7:**
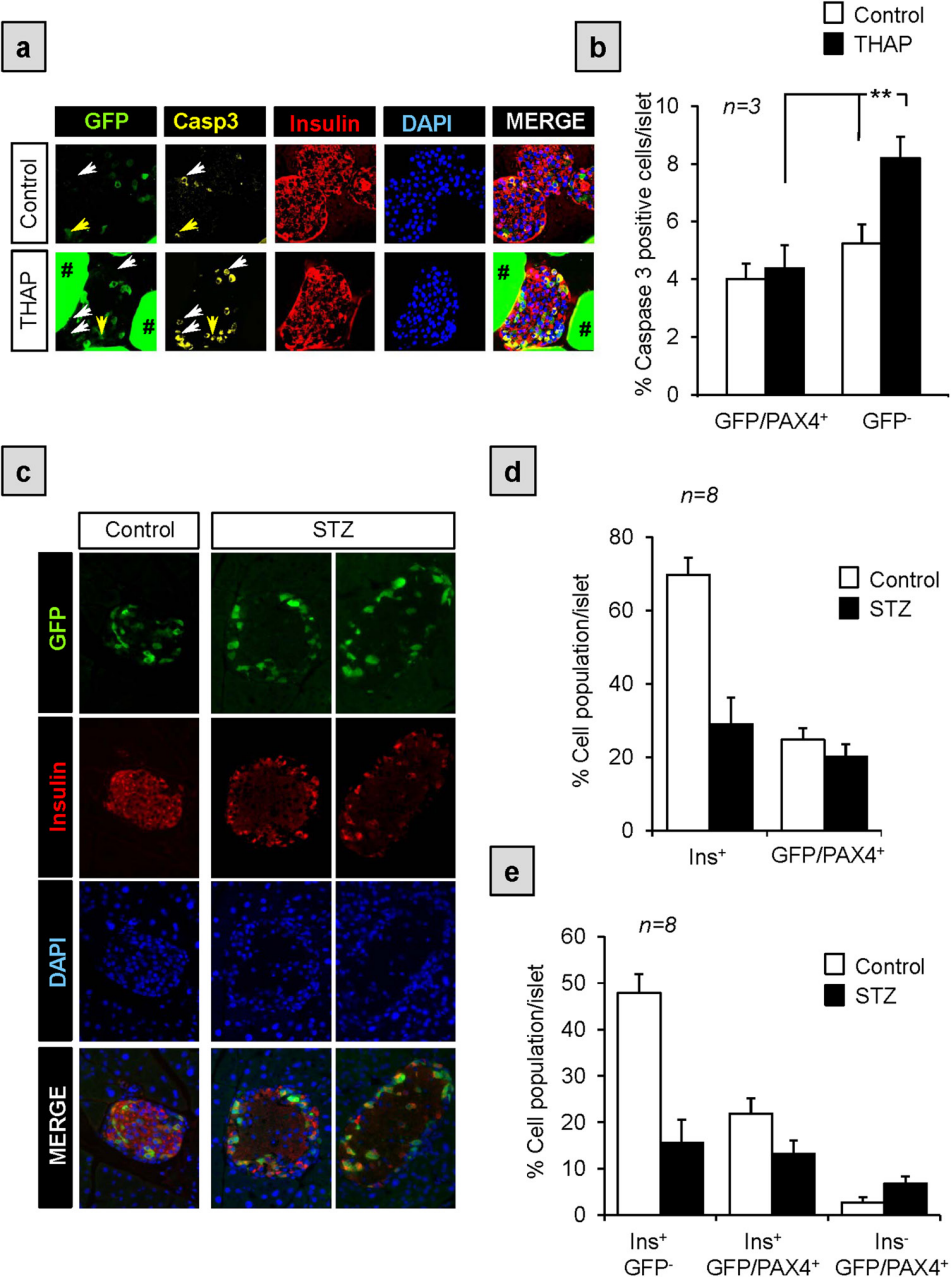
GFP/PAX4^+^ cells are more resistant to apoptosis than their GFP/PAX4^−^ counterparts. (**a**) Immunofluorescent detection of GFP (green), cleaved-CASPASE 3 (yellow), and INSULIN (red) as well as DAPI nuclear staining (blue) in islets isolated from pPax4-*Cre*-IRES-*Egfp* and treated *in vitro* for 24 hours with thapsigargin (THAP). Arrows depict examples of GFP^−^/cleaved caspase 3^+^ cells. Non-specific GFP labeling of Affi-Gel® beads used for islet mounting is denoted by #. (**b**) Cell quantification represented as the percentage of cleaved caspase 3-positive cells in either GFP/PAX4^+^or GFP^−^ cells treated or not with thapsigargin. Data show the mean ± SE of 3 independent experiments, each representing at least 10 islets and 1000 cells per conditions. **ANOVA:p < 0.01. (**c**) pPax4-*Cre*-IRES-*Egfp* mice were injected (*i.p*.) with STZ (200 mg/kg body weight) to promote β-cell apoptosis and pancreas extracted 24 hours post treatment. Animals were normoglycemic at this time point. Pancreas sections were then co-immunostained for GFP (green) and INSULIN (red). (**d**) The number of INSULIN^+^ and GFP/PAX4^+^cells were counted and results presented as the percentage of total islet cells. (**e**) Percentage of GFP^−^ and GFP/PAX4^+^ cells between INSULIN^+^ and INSULIN^−^ cells. Data show the mean ± SE of 8 independent animals, each representing an average of 15 islets and 1500 cells per animal.

High levels of PAX4 were also shown to protect β-cells against stress-induced apoptosis as well as to act as a survival factor in INS-1E insulinoma cells^2^,^15^,^17^. This PAX4-mediated protective effect was recently correlated to improve calcium and endoplasmic reticulum (ER) homeostasis (Mellado-Gil *et al.*, manuscript submitted). This data led us to postulate that GFP/PAX4^+^ cells within islets would be more resistant to apoptosis as compared to their GFP^−^ counterpart. To validate this assumption, islets isolated from pPax4-*Cre*-IRES-*Egfp* mice were cultured in the presence of thapsigargin (THAP), an inhibitor of ubiquitous ER Ca^2+^-ATPases that induces ER-stress dependent apoptosis. Cleaved-CASPASE-3 immunostaining revealed a significant 2-fold enrichment in apoptotic GFP^−^ cells within THAP-treated islets as compared to untreated controls, while no significant increase in apoptosis was discerned in GFP/PAX4^+^ cells (Fig.7a,b). In order to substantiate these findings in a more physiological context, pPax4-*Cre*-IRES-*Egfp* mice were treated with streptozotocin (STZ), an agent that specifically destroys β-cells. Animals were sacrificed 24 hours post-STZ treatment prior to the development of hyperglycemia with the aim to clearly discriminate the impact of STZ on the two GFP subpopulations. As anticipated, the number of GFP/PAX4^+^ cells remained relative constant while the overall β-cell population drastically decreased from 70 to 30% of all islet cells in STZ-treated mice (Fig. 7c,d). A more detailed analysis of the various β-cell populations based on GFP expression pattern revealed that the decrease in β-cell mass predominantly stemmed from the loss of GFP^−^cells while GFP/PAX4^+^β-cells were refractory to STZ-mediated destruction (Fig. 7e). Of note, we did not observed any gender specific effect of STZ on the two distinct subpopulations. However, a slight increase in the percentage of INSULIN^−^ cells in the GFP/PAX4^+^ subpopulation was discerned upon STZ treatment (Fig. 7e) potentially as a result of either re-expression of PAX4 in α-cells or loss of β-cell entity. Immunofluorescence analysis revealed that the percentage of GFP/PAX4^+^ cells among glucagon expressing cells remained relatively constant after STZ treatment (Supplementary Fig. S2) suggesting that INSULIN^+^/GFP/PAX4^+^ cells were undergoing de-differentiation resulting in the loss of INSULIN expression.

## Discussion

Our work establishes the existence of a *bona fide* PAX4-enriched cell subpopulation nested within pancreatic islets that can, on one hand expand in periods of increased functional demands, and on the other hand exhibit improved viability in response to pathophysiological situations. These results foster the prospect that PAX4, a critical regulator of embryonic β-cell development, maintains plasticity within a pool of cells in adult islets concealed predominantly as β-cells. These cells are dispersed throughout the islet without any apparent niche. Heterogeneity among islet β-cells has been well documented and first described by Pipeleers as the sociobiology of pancreatic β-cells^28^. Consistent with the notion of an islet β-cell progenitor reservoir contributing to this heterogeneity, several studies using reporter constructs to track the fate of β-cells either *in vitro* or *in vivo*, revealed the existence of at least three distinct subpopulations of β-cells within human and mouse islets^29^,^30^,^31^. Of particular interest was the characterization of a proliferative immature PDX1^+^/INSULIN^low^ β-cell subpopulation that comprised approximately 15 to 25% of all islet cells and that expressed higher levels of PAX4^32^. This cell population is reminiscent to that of PDX1^+^/INSULIN^low^/BrdU^+^ β-cells characterized in PAX4 over-expressing mice^17^ as well as to the endogenous GFP/PAX4^+^ cell fraction described herein. Nonetheless, GSIS was normal in GFP/PAX4^+^ cells whereas it was impaired in both PAX4 over-expressing islets and in the reported PDX1^+^/INSULIN^low^ β-cell subpopulation^17^,^32^. As the insulin content of both GFP/PAX4^+^ and GFP^−^ cells was identical (as assessed for GSIS presented in Fig. 1g), we propose that pending environmental cues expression levels of PAX4 will define the fate, state and functionality of endogenous islet cells. Such premise is reinforced by the fact that some of the GFP/PAX4^+^ cells express SOMATOSTATIN, GLUCAGON or INSULIN and GLUCAGON together, resembling embryonic cell fate commitment process, where dual hormone cells have been detected^33^,^34^.

Our data reconcile divergent views regarding an ongoing debate on whether β-cell expansion involves an elusive progenitor cell pool or proceeds through self-duplication of resident β-cells^35^,^36^,^37^. Consistent with self-duplication advocates, we find that under normal conditions, both GFP/PAX4^+^and GFP^−^ β-cells are prone to proliferation, a state that is gradually impaired with age^38^. However, in physiological situation evoking an increase in the β-cell mass, as during pregnancy, we witnessed a specific enrichment in the replication of GFP/PAX4^+^ cells as compared to the GFP^−^ cells advocating in favor of the recruitment of a specialized plastic β-cell pool. This specific mobilization could be a consequence of GFP/PAX4^+^ cells being permissive to cell proliferation as revealed by increased cell cycle genes in equivalent PAX4-overexpressing islet cells. A similar cell cycle re-entry dependent permissive state was recently reported for islets conditionally overexpressing the connective tissue growth factor^39^. Hence, our study demonstrates that both GFP/PAX4^+^ and GFP^−^ β-cells are susceptible to basal proliferation but that only the fraction, defined by expression levels of PAX4, are permissive to increased replication pending stimuli. However, this capacity to proliferate declines with age as assessed by both a decrease in the GFP/PAX4^+^ subpopulation as well as in cell replication correlating with increased failure to adapt β-cell mass with age^40^.

Additional members of the *pax* gene family have been shown to convey similar proliferative and survival phenotypes within adult tissues. For example, PAX6 is essential for the production and subsequent maintenance of progenitor cells in the adult hippocampal dentate gyrus while PAX7 expression is critical for survival and plasticity of adult skeletal muscle satellite cells in response to environmental cues^41^,^42^. In addition, PAX3 expressing melanocytic progenitor cells are permissive to injury signals for re-entry into cell cycle and initiate regeneration whereas PAX2 re-activation is essential for kidney injury repair^43^,^44^. Interestingly, *pax* genes have arisen from a single gene during the early metazoan era emphasizing a common ancestral function among the *pax* members including PAX4. This function that pre-dates gene duplication most likely relates to the capability of expressing host cells to respond to a dynamic environment^45^. The latter also includes increased survival in response to hostile settings, a premise also demonstrated by the ability of the GFP/PAX4^+^ cell subset to preferentially resist STZ and thapsigargin-mediated apoptosis as compared to GFP^−^ cells.

Recent studies suggest that in the diabetic state β-cells revert to NGN3^+^/MAFA^−^/INSULIN^−^ progenitor-like cells rather than undergoing massive apoptosis^18^,^19^,^46^. Re-differentiation of dedifferentiated cells was possible following insulin therapy^19^. Interestingly, we previously demonstrated that PAX4 transcript levels are induced by high glucose and are dramatically increased in islets obtained from T2DM donors correlating with increased survival^2^,^15^. Chronic PAX4 expression was also shown to lead to MAFA and INSULIN repression reminiscent of the progenitor-like cells induced by the diabetic milieu^19^. Of particular interest is the finding that NGN3 was increased in islet overexpressing PAX4. Taken together, it is tempting to speculate that under pathophysiological conditions, acute increased in PAX4 expression will initially protect cells while sustained expression will induce dedifferentiation towards a ‘progenitor phenotype’ as an adaptive mechanism to protect cells against a hostile environment. In support of this viewpoint, we observed a small increase in INSULIN^−^ cells within the GFP/PAX4^+^ subpopulation subsequent to STZ treatment (Fig. 7e) while others have reported robust increases in PAX4 expression levels in islets of STZ-treated mice^47^. Once the threat is neutralized, repression of PAX4, by mechanisms independent of DNA methylation, will promote re-differentiation towards a β-cell phenotype.

*Pax4* could be considered a ‘selfish gene’ safeguarding survival of the fittest cell population within islets^48^. Indeed, by exquisitely fine tuning its levels in response to the microenvironment, PAX4 will act as a rheostat in defining the phenotypic characteristic of islet cells to best adapt to their new surroundings. Functional impairment associated with PAX4-dependent re-entry into cell cycle would have little consequence short term on glucose homeostasis, as the predominant β-cell mass would remain quiescent and functional. Disparity in the impact of *pax4* gene mutations on the incidence of diabetes could potentially be rationalized by the outcome of gene-environment interaction, which will dictate fitness of β-cells. In conclusion, strategies to fine-tune PAX4 expression levels may constitute a promising approach to simultaneously expand while blocking dedifferentiation of the functional β-cell mass with the aim to salvage hyperglycemia in diabetic patients.

## Methods

### Animals

Mice were maintained on a normal light/dark cycle and studied under conditions approved by the Institutional Animal Care Committee of CABIMER. Methods associated with animals were carried out in accordance with the approved guidelines.The pPax4-*Cre*-IRES-*Egfp* mice, kindly provided by Dr. P. Gruss (Max Planck Institute, Gottingen, DE), were back crossed to C57BL/6 mice (Jackson Laboratory). Animals were genotyped with the REDExtract-N-Amp kit (Sigma-Aldrich, Madrid, Spain) using specific primers that can be obtained upon request. Lineage tracing experiments were conducted on the progeny of pPax4-*Cre*-IRES-*Egfp* mice bred to *Rosa26*;lox-stop-lox;*LacZ* mice. For *in vivo* cell death analysis, adult (3–4 months) pPax4-*Cre*-IRES-*Egfp* mice (average body weight 25 g) were treated with a single high-dose of streptozotocin (STZ: 200 mg/kg body weight) by intraperitoneal injection. STZ was dissolved in 0.1 M sodium citrate buffer and injected within 5 min of dissolution. Blood glucose levels from tail vein samples were determined prior to and 24 hours after STZ injection using a Precision Xceed glucometer (Abbott, Madrid, Spain).

### Mouse Islet Isolation and Treatment

Mouse pancreatic islets were isolated by intraductal collagenase as described elsewhere^17^. Islets were maintained overnight in 11.1 mM glucose/RPMI-1640 (Life Technologies, Madrid, Spain) supplemented with 10% fetal bovine serum (Sigma), 100 Units/ml penicillin and 100 μg/ml streptomycin (Sigma), 2 mM glutamine (Life Technologies), 10 mM hepes (Life Technologies), 1 mM sodium pyruvate (Sigma), and 50 μM β-mercaptoethanol (Life Technologies). In some instances, GFP fluorescence from living islets was captured using and Image-Xpress device (Molecular devices, Spain). Composition of the various islet cell subpopulations was assessed by flow cytometry (FACSCalibur, BD Biosciences) while isolation of GFP^+^ and GFP^−^ islet cells was achieved using FACS (FACSAria,BD Biosciences) and FACSDiva software (BD Biosciences). Briefly, isolated islets from adult (3–4 months) C57BL/6 WT mice and pPax4-*Cre*-IRES-*Egfp* mice were dispersed by trypsinization, resuspended in PBS containing 25 mM Hepes and 2.5 mM EDTA and filtered through cell strainer caps (70 μm). Using fluorescence-activated sorting, β-cells were separated from non-β-cells based on their size (FSC channel) and autofluorescence (FL-1 channel-GFP), as previously described^49^. Purified subpopulations were plated on an 804G matrix rich in laminin for 24 hours to improve cell viability prior to insulin secretion assay. The latter was performed by incubating cells for 30 min in Krebs-Ringer bicarbonate Hepes buffer containing 2.5, 16.5 mM glucose or a mixture of 16.5 mM glucose/1 μM forskolin/100 μM isobutylmethylxanthine (IBMX). Supernatants were collected and cells lysed in 1 mM acetic acid/ethanol. Secreted and total cellular content of insulin were assessed using an ELISA kit (Linco, St Charles, MO). For *in vitro* thapsigargin-induced cell death assessment, groups of 150 islets were incubated or not during 24 hours with 1 μM thapsigargin. Islets were then embedded according to the protocol of Cozar-Castellano *et al.*^50^ and processed for IHC analysis.

### RNA Isolation and Quantitative PCR

RNA was extracted from GFP^+^ and GFP^−^ islet cells using the RNeasy micro Plus kit (Qiagen Iberia S.L., Madrid Spain). After quantification and analysis of the RNA integrity using a 2100 bioanalyzer (Agilent-Eukaryote Total RNA Nano Series), 100 to 200 ng RNA was used for cDNA synthesis using Super Script II RT (Life Technologies) and Anchored OligodT as primers. For RNA extraction from whole islets, RNeasy micro Plus kit (Qiagen Iberia S.L., Madrid Spain) was used. Between 800 ng and 1 μg of RNA was employed for cDNA synthesis using Super Script II RT (Life Technologies) and Anchored OligodT as primers. Quantitative PCR was performed using a 7500 Real-Time PCR System (Applied Biosystems). Specific primers for the analyzed genes (Pax4, Pdx1 and Insulin) were designed to span an intron in order to avoid genomic DNA amplification. The housekeeping genes β-actin and cyclophilin were used as control.

### RNA Microarray

Labeled cRNA samples were prepared from pools of at least 100 islets isolated from Pax4/rtTA transgenic animals (8-week old females) treated or not with DOX as previously described^17^. Three independent preparations of cRNA per group were then hybridized to the GeneChip Mouse Gene 1.0 ST Array chip (Affymetrix, Santa Clara, CA) using standard protocols of the Genomic Core Facility of CABIMER. Analysis of the transcriptome profiling is described elsewhere (Mellado Gil *et al.*, Manuscript submitted). Raw data are accessible in the Gene Expression Omnibus database under accession number GSE62846.

### Bisulfite Sequencing

DNA was extracted from MIN6 cells as well as from FACS purified non-β, GFP^+^ and GFP^−^ subpopulations. Bisulfite conversion of unmethylated C to U was performed using “Cells-to-CpG Bisulfite Conversion” kit (Life Technologies) following guidelines of the manufacturer. Converted DNA was amplified using MyTaqHS DNA polymerase (Bioline, Paris, France) along with specific primers for the region of interest on the *pax4* gene promoter. Amplicons were purified using QIAquick PCR purification kit (Qiagen Iberia S.L.), cloned into the pGEMT vector and transformed into DH5α *E. Coli* bacteria. At least 20 colonies per DNA region and cell subpopulation were sequenced and analyzed using the BiQ Analyzer software.

### Immunohistochemistry

For paraffin sections, pancreata were dissected and fixed overnight in 4% paraformaldehyde at 4 °C. Dehydration, embedding, and sectioning at 5 μm thickness were performed by the Histology platform of CABIMER. Sections were rehydrated in decreasing ethanol concentration solutions and subjected to heat-induced antigen retrieval in 10 mM sodium citrate buffer pH 6. Sections were then blocked in PBS containing 5% donkey serum and 0.2%TritonX100 for 1 h at room temperature. Primary antibodies were incubated overnight at 4 °C in PBS 1% BSA 0.2% TritonX100, except for GFP antibody that was incubated in PBS 0.1% BSA 0.2% TritonX100. The following primary antibodies and dilutions were used: Goat anti-GFP 1:200 (Abcam), mouse anti-INSULIN 1:500 (Sigma), rabbit anti-INSULIN 1:100 (Santa Cruz), mouse anti-GLUCAGON 1:150 (Sigma), mouse anti-PDX1 1:150 (Hybridoma bank), rabbit anti-SOMATOSTATIN 1:150 (Santa Cruz), goat anti-SOMATOSTATIN 1:100 (Santa Cruz), mouse anti-E-CADHERIN 1:250 (BD Transduction laboratories), mouse anti-Ki67 1:150 (Novocastra), rabbit anti-Ki67 1:150 (Pierce-Thermo Scientific), rabbit anti-β-GAL1:500 (MP Biomedicals) and rabbit anti-cleaved CASPASE-3 1:150 (Cell Signaling). Secondary antibodies were incubated for 1 hour at room temperature at a 1:800 dilution in PBS 0.2% TritonX100. The following secondary antibodies were employed: Alexa Fluor 488 donkey anti-goat, Alexa Fluor 555 donkey anti-mouse and Alexa Fluor 647 donkey anti-rabbit (Life Technologies). Counterstaining was performed with 5 μg/ml DAPI (Life Technologies) and slides finally mounted using fluorescent mounting medium (DAKO). Epifluorescence microscopy images were taken with a Leica DM6000B microscope. For image quantification, slides from 4 to 8 animals per time point were studied with an average of 15 islets per animal.

### Statistical Analysis

Results are expressed as mean ±  SE. Statistical differences were estimated using a 2-tailed Student’s test for comparison between 2 groups and 1-way ANOVA for more that 2 groups, with Scheffe’s F test for post-hoc analysis.

## Additional Information

**How to cite this article**: Lorenzo, P. I. *et al.* Pax4 Defines an Expandable β-Cell Subpopulation in the Adult Pancreatic Islet. *Sci. Rep.* **5**, 15672; doi: 10.1038/srep15672 (2015).

## Supplementary Material

Supplementary Information

## Figures and Tables

**Table 1:** Significantly up-regulated cell cycle genes after Pax4 overexpression.

Symbol	Gene Description	fold change	p value
**Errfi1**	ERBB receptor feedback inhibitor 1	2.254	0.020
**Cdkn1a**	cyclin-dependent kinase inhibitor 1A (p21)	1.956	0.011
**Ccnb2**	cyclin B2	1.478	0.012
**Ccna2**	cyclin A2	1.447	0.001
**Myc**	myelocytomatosis oncogene	1.412	0.003
**Cdk5**	cyclin-dependent kinase 5	1.368	0.002
**Trp53**	transformation related protein 53	1.361	0.013
**Ccnd3**	cyclin D3	1.339	0.034
**Ccnd1**	cyclin D1	1.275	0.026
**Hdac1**	histone deacetylase 1	1.272	0.042
**Pcna**	proliferating cell nuclear antigen	1.271	0.032
**Otub2**	OTU domain, ubiquitin aldehyde binding 2	1.262	0.047
