# Supplementary material for: PAX4 Defines an Expandable β-Cell Subpopulation in the Adult Pancreatic Islet
Source: Sci Rep. 2015 Oct 27;5:15672. doi: 10.1038/srep15672 (PMC4622080; doi:10.1038/srep15672)
Supplement: Supplementary Information [file srep15672-s1.pdf]

SUPPLEMENTARY INFORMATION

Pax4 Defines an Expandable β-Cell Subpopulation in the Adult Pancreatic Islet

Petra I. Lorenzo[1,*], Esther Fuente-Martín[1], Thierry Brun[2], Nadia Cobo-Vuilleumier[1], Carmen María Jimenez-Moreno[1], Irene de Gracia Herrera Gomez[1], Livia López Noriega[1], José Manuel Mellado-Gil[1], Alejandro Martin-Montalvo[1], Bernat Soria[3, 4] and Benoit R. Gauthier[1,*]

[1]Pancreatic Islet Development and Regeneration Unit and [3]Cellular Therapy of Diabetes Mellitus and its Complications, Department of Stem Cells, CABIMER-Andalusian Center for Molecular Biology and Regenerative Medicine, Seville, Spain; [2]Department of Cell Physiology and Metabolism, University of Geneva, Geneva, Switzerland and [4]CIBERDEM, Instituto Carlos III, Madrid, Spain

* Corresponding authors:

Benoit R. Gauthier, PhD. e-mail: benoit.gauthier@cabimer.es

Petra I. Lorenzo, PhD. e-mail: petra.lorenzo@cabimer.es

Pancreatic Islet Development and Regeneration Unit
Department of Stem Cells, CABIMER
Av. Américo Vespucio, Parque Científico y Tecnológico Cartuja 93
41092 Sevilla

SUPPLEMENTARY FIGURE S1: Lorenzo, P.I. *et al.*

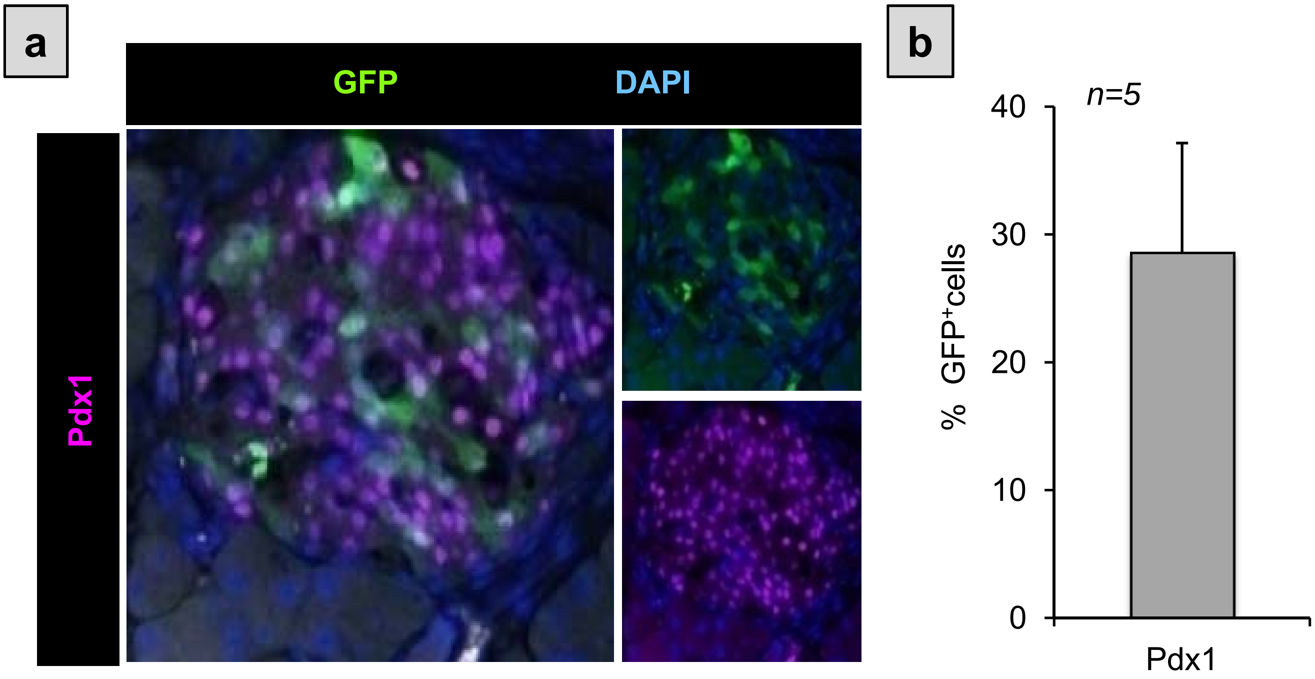

Supplementary Figure S1. GFP expression predominantly co-localizes with β-cells in adult islets. **a)** Immunohistochemistry analysis of paraffin sections from adult pPax4-*Cre*-IRES-*Egfp* mice pancreas. Representative microscopy images for co-immunofluorescent labeling of GFP (green) with the β-cell marker PDX1 (pink). Nuclei counterstaining was performed using DAPI (blue). **b)** Quantification of the percentage of Pdx1$^+$ cells that co-express GFP (average ± SE). Sections from 5 animals with an average of 15 islets and 1400 cells per sections were used for quantifications.

SUPPLEMENTARY FIGURE S2: Lorenzo, P.I. *et al.*

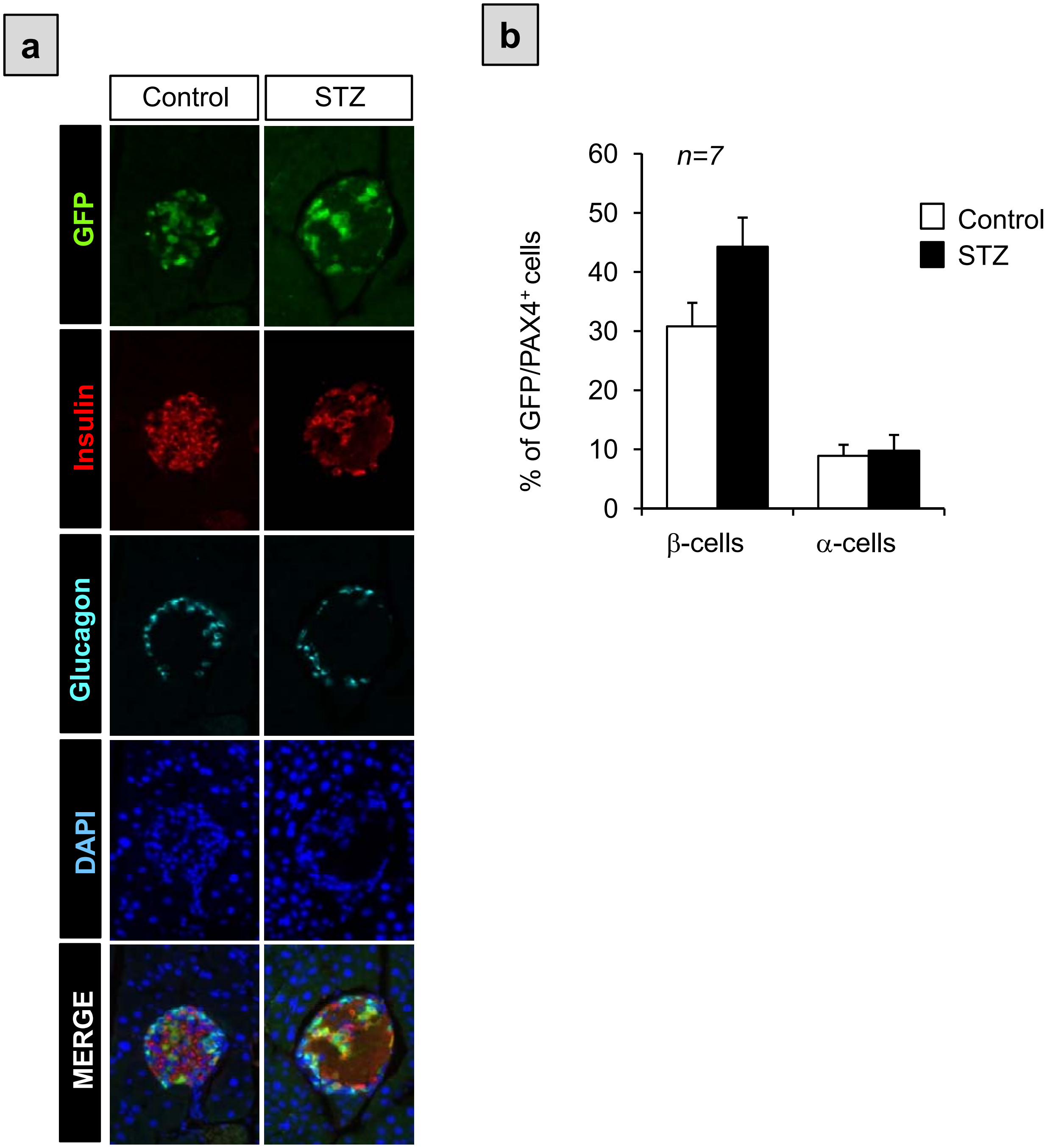

Supplementary Figure S2. Pax4 is not re-expressed in α-cells. pPax4-*Cre*-IRES-*Egfp* mice were injected (*i.p.*) with STZ (200mg/kg body weight) to induce β-cell apoptosis and pancreas extracted 24 hours post treatment. **a)** Immunofluorescent detection of GFP (green), INSULIN (red), and GLUCAGON (cian) as well as DAPI nuclear staining (blue) in pancreas sections of STZ treated and non-treated (Ctrl) animals. **b)** Quantification of the percentage of GFP/PAX4$^+$ cells among insulin$^+$, or glucagon$^+$ cells (average ± SE). Sections from 7 animals with an average of 10 islets and 1000 cells per sections were used for quantifications.
